# Niche-dependent development of functional neuronal networks from embryonic stem cell-derived neural populations

**DOI:** 10.1186/1471-2202-10-93

**Published:** 2009-08-06

**Authors:** Sebastian Illes, Stephan Theiss, Hans-Peter Hartung, Mario Siebler, Marcel Dihné

**Affiliations:** 1 Department of Neurology, Heinrich-Heine University, Moorenstr. 5, 40225 Düsseldorf, Germany

## Abstract

**Background:**

The present work was performed to investigate the ability of two different embryonic stem (ES) cell-derived neural precursor populations to generate functional neuronal networks *in vitro*. The first ES cell-derived neural precursor population was cultivated as free-floating neural aggregates which are known to form a developmental niche comprising different types of neural cells, including neural precursor cells (NPCs), progenitor cells and even further matured cells. This niche provides by itself a variety of different growth factors and extracellular matrix proteins that influence the proliferation and differentiation of neural precursor and progenitor cells. The second population was cultivated adherently in monolayer cultures to control most stringently the extracellular environment. This population comprises highly homogeneous NPCs which are supposed to represent an attractive way to provide well-defined neuronal progeny. However, the ability of these different ES cell-derived immature neural cell populations to generate functional neuronal networks has not been assessed so far.

**Results:**

While both precursor populations were shown to differentiate into sufficient quantities of mature NeuN^+ ^neurons that also express GABA or vesicular-glutamate-transporter-2 (vGlut2), only aggregate-derived neuronal populations exhibited a synchronously oscillating network activity 2-4 weeks after initiating the differentiation as detected by the microelectrode array technology. Neurons derived from homogeneous NPCs within monolayer cultures did merely show uncorrelated spiking activity even when differentiated for up to 12 weeks. We demonstrated that these neurons exhibited sparsely ramified neurites and an embryonic vGlut2 distribution suggesting an inhibited terminal neuronal maturation. In comparison, neurons derived from heterogeneous populations within neural aggregates appeared as fully mature with a dense neurite network and punctuated vGlut2 expression within presynaptic vesicles. Also those NPCs that had migrated away from adherent neural aggregates maintained their ability to generate a synchronously oscillating neuronal network, even if they were separated from adherent aggregates, dissociated and re-plated.

**Conclusion:**

These findings suggest that the complex environment within niches and aggregates of heterogeneous neural cell populations support the generation of fully mature neurons and functional neuronal networks from ES cell-derived neural cells. In contrast, homogeneous ES cell-derived NPCs within monolayer cultures exhibited an impaired functional neuronal maturation.

## Background

Functional biological neuronal networks *in vitro *can be derived from primary tissue-derived neurons and represent populations of synaptically interconnected cells capable of generating synchronous electrophysiological activity [1-6]. Electrophysiological recordings of entire neuronal networks can be achieved by microelectrode arrays (MEAs) which can detect electrophysiological activity of spatially distributed neuronal populations by multi-electrode recordings over several weeks or months [5,7-10]. *In vitro *generated neuronal networks that represent the basic principle for brain activity can be used to analyze the electrophysiological development and quality of interconnected neuronal populations of different cell sources as well as their reactions to pharmacologically active compounds. The formation of functional neuronal networks requires fundamental properties of neural populations such as the ability to exhibit adequate neurite outgrowth, synaptogenesis, neurotransmitter production and release, action potentials as well as the generation of glial cells supporting neuronal functions. If only one of these fundamental properties is absent, the function of neuronal networks might be heavily impaired or even entirely blocked.

Recently, we and others demonstrated that also embryonic stem (ES) cell-derived neural precursor populations are able to generate functional neuronal networks [11,12]. We illustrated that the development of an ES cell-derived neuronal network is characterized by distinct stages of specific electrophysiological activity that finally results in spatially distributed synchronously oscillating bursts [11]. The rationale for using ES cell-derived instead of primary tissue-derived neural populations is the increased homogeneity and controllability of *in vitro *generated functional neuronal networks whose reactions, for instance, to different pharmacological compounds strongly depend on the standardization of the cell culture. Furthermore, the possibility of measuring the electrophysiological function of *in vitro *generated neuronal networks permits to verify the quality of different precursor-derived neuronal populations prior to transplantation within the scope of regenerative medicine.

In recent years, the so called niche-independent development of neural precursor cells in monolayer cultures instead of precursor development within niches of aggregated cells of different developmental stages, also called neurospheres, was regarded as the most efficient way to synchronize and control neural precursor cell development *in vitro*. In order to improve standardization and homogeneity of ES cell-derived neuronal networks, we also applied the niche-independent protocols [13] for monolayer neural precursor cells (MNPs) that were generated initially from serum-free, floating cultures of embryoid body-like aggregates (SFEBs). We were able to produce highly homogeneous cultures of MNPs that adopted a radial glia-like cell stage and differentiated under controlled conditions into astrocytes and oligodendrocytes as well as mature NeuN^+ ^neurons that also expressed GABA or vesicular-glutamate-transporter-2 (vGlut2). While dense MNP cultures, differentiated for at least 28 days, were able to generate spontaneously spiking neurons as expected, they were, however, not able to generate functional, synchronously oscillating neuronal networks as demonstrated by the MEA technology. Instead, we could show that the development of functional neuronal networks was reliably possible when heterogeneous neural cell populations derived from aggregated neural precursor cell-enriched SFEB (nSFEB) niches were used. As comparison of the neuronal morphology between MNP- and nSFEB-derived neuronal populations revealed shortcomings of MNP-derived neurons with respect to neurite morphology and maturity of glutamatergic neurons, we speculate that heterogeneous neural cell populations within neural aggregates provide factors which support the generation of functional neuronal networks.

## Methods

### Neural differentiation of ES cells

The ES cell lines SV-129 (ATCC, Millipore, Germany), R1 [14] and an ES cell line constitutively expressing enhanced green fluorescent protein (EGFP) derived from transgenic C57BL/6J mice ubiquitously expressing EGFP under the influence of the chicken β-actin promoter [15] were used in this study. Undifferentiated ES cells were grown under feeder-deprived conditions in the presence of 1,000 U/ml leukemia inhibitory factor (LIF, Chemicon, Temecula, CA, USA) and 20% fetal bovine serum (FBS, HyClone, Thermo Fisher Scientific, Germany) in ES cell medium described elsewhere [16]. Neural differentiation of immature ES cells was performed by modified protocols according to Watanabe (2005) and Nat (2007). Briefly, ES cell colonies were harvested without dissociation and re-plated on bacterial dishes (Greiner, Germany) in ES cell medium containing 10% FBS without LIF [17]. After one day, free-floating embryoid bodies had developed and were re-plated in neural induction medium (NIM) consisting of DMEM/F12 and neurobasal medium (1:1) (both from Gibco-BRL), 2 mM Glutamax (Gibco-BRL) and N2 supplement according to Johe and colleagues [18] as well as B27 supplement (Gibco-BRL). After 7 days, serum-free, floating cultures of embryoid body-like aggregates (SFEB) had developed consisting predominantly of neural precursor cells [19-21]. SFEBs were used to generate neural precursor cell-enriched SFEBs (nSFEBs) or adherently growing monolayer neural precursors (MNP).

For the generation of nSFEBs, free-floating SFEBs were cultivated for 7 to 14 days in the presence of neural proliferation medium (NPM) consisting of DMEM/F12, 2 mM Glutamax, fibroblast growth factor-2 (FGF-2), epidermal growth factor (EGF) (20 ng/ml each, PeproTech) and N2 supplement.

For the generation of secondary neural cell populations derived from nSFEBs, nSFEBs were plated on laminin coated dishes and kept under the influence of EGF and FGF-2 for 5 to 7 days. During that time a dense cell layer of mixed neural cells had spread out from the nSFEB aggregates. Entire populations including the compact nSFEB aggregates and the population that had spread out were detached by accutase (Chemicon, Germany) treatment for 20 minutes at 37 degrees, slightly triturated while leaving the nSFEB aggregates intact and then transferred to conical tubes and allowed to sediment for 10 minutes. Single cells within the supernatant, referred to as secondary neural cell populations derived from nSFEBs (SNPs), were collected and transferred on PDL/laminin-coated substrates for further characterization.

For the generation of MNPs, SFEBs were seeded on gelatin (0,1%, Sigma-Aldrich) coated plastic dishes (Greiner) in the presence of neural stem cell expansion medium (NS-A medium, Euroclone, Milan, Italy) supplemented with modified N2 and 20 ng/ml of both EGF and FGF-2. After 4-6 days in culture, cells migrating away from attached SFEBs were selectively enriched by multiple passages according to Conti and colleagues (2005).

### Differentiation of nSFEBs, SNPs and MNPs

For propagation, free-floating nSFEBs were passaged once a week under the influence of EGF and FGF-2. MNPs were cultivated on gelatin coated dishes in NS-A medium under the influence of EGF and FGF-2.

For differentiation, nSFEBs (5-10 aggregates/100 μl), dissociated SNPs or MNPs (40.000 cells to 80.000 cells/100 μl) were seeded on poly-D-lysine (PDL, 15 μg/ml, Sigma) and laminin (15 μg/ml, Sigma) - coated glass coverslips or microelectrode arrays (Multi Channel Systems, Reutlingen, Germany). For successful long-term attachment of cells on MEAs, PDL coating was performed for 1 day and laminin coating for 5 days at 4°C. To improve cell survival and induce a latent neurogenic program [22], cells were kept for further 6-10 days after plating under the influence of FGF-2 only, before also this growth factor was gradually removed from the medium to induce terminal differentiation. For long-term culture, ES cell-derived neuronal networks were kept in DMEM/F12 supplemented with N2, B27 and Glutamax.

### Immunocytochemistry and quantification

For immunocytochemical investigations, cultured cells were washed in phosphate-buffered saline (PBS), pH 7.3 and fixed for 15 min in 4% paraformaldehyde before incubation with 1% normal goat serum (NGS, Sigma) in PBS for one hour. Primary antibodies were monoclonal mouse antibodies to βtubulin III (1:750, R&D Systems), glial fibrillary acidic protein (GFAP, 1:1000, DAKO) nestin (1:750, Chemicon), myelin basic protein (MBP, 1:1000, Chemicon), NeuN (1:500, Chemicon), synaptophysin (1:100, Sigma), GABA (1:1000, Sigma) and vesicular glutamate transporter 2 (vGlut2, 1:10000, Chemicon). They were applied at 4°C over night. After washing in PBS, appropriate secondary antibodies coupled to Cy2, Cy3 or Cy5 (1:750, Dianova, Hamburg, Germany) were applied for one hour at room temperature. Cell cultures were counterstained for one minute with 4',6-Diaminodino-2-Phenylindol (DAPI, 2 μg/ml, Serva) to visualize cell nuclei. Images were collected with a confocal-laser scanning microscope (LSM 510 Zeiss, Germany).

For quantification of neurons, astrocytes or oligodendrocytes, percentages of NeuN^+^, GFAP^+ ^or MBP^+ ^cells from all DAPI^+ ^cells were counted with "Nucleolus Counter Plugin" and "Cell Counter Plugin" provided by ImageJ software.

### Microelectrode array recordings

To measure the electrophysiological activity of nSFEB-, SNP- and MNP-derived neurons, microelectrode arrays (MEAs) were used with a square grid of 60 planar Ti/TiN electrodes (30-μm diameter, 200-μm spacing) and an input impedance of <50 kÙ according to the specifications of the manufacturer (Multi Channel Systems, Reutlingen, Germany). Signals from all 60 electrodes were simultaneously sampled at 25 kHz, visualized and stored using the standard software MC_Rack provided by Multi Channel Systems. Spike and burst detection was performed off-line by custom-built software (Result, Düsseldorf, Germany). Individually for each channel, the threshold for spike detection was set to 6.2 standard deviations (SDs) of the average noise amplitude during a 10% "learning phase" at the beginning of each measurement. An absolute refractory period of 4 ms and a maximum spike width of 2 ms were imposed on the spike detection algorithm. All spike waveforms were stored separately and visually inspected for artifacts. Burst detection was performed individually for each electrode by comparing the actual temporal clustering of spikes with a Poisson process of independently occurring spikes at the same mean firing rate (null hypothesis). Given the mean firing rate (MFR) of each electrode, the probability P(N, Δt|MFR) of finding at least N spikes within the time span Δt was calculated using the Poisson distribution. An observed cluster of N>3 spikes occurring within Δt was considered as burst, if its Poisson probability P(N, Δt|MFR) was below 0.005. Note that although bursts frequently occurred as population events, i.e. synchronized across many electrodes, they were detected separately for each electrode allowing for different mean firing rates. Bursts were also verified for plausibility by visual inspection. For the quantification of firing synchrony across two electrodes spikes were collected in 10 ms wide bins. Bins were then dichotomized to contain either zero spikes or at least one spike. For two electrodes (1, 2) with s_1 _(s_2_) bins containing spikes on electrode 1 (2), c_12 _bins with coincident spikes on both electrodes, and a total of N bins in the recording, there is an expected proportion p_exp _= ((N - s_1_)·(N - s_2_) + s_1_·s_2_)/N^2 ^of coincident bins (both either empty or with coincident spikes), if firing occurred independently on both electrodes. Cohen's kappa statistic κ then captures the excess part of the proportion of observed coincident bins p_obs _over the expected proportion p_exp_: p_obs _= p_exp _+ κ·(1-p_exp_) [23]. Kappa values lie in the range -p_exp_/(1-p_exp_) to +1. The average kappa of all electrode pairs with a firing rate of at least 50 spikes per minute was calculated as measure of overall synchrony of a recording.

### Statistical analysis

MEA recordings for the different groups (nSFEB, SNP, MNP) were repeated at least 10 times for each group with at least 3 independent cell cultures. To verify electrophysiological differences of aggregates, clusters and interjacent cells, at least 826 electrodes, respectively, were ascribed to the spatially different compartments of individual cultures and electrophysiologically analyzed. Data were analyzed with one way analysis of variance (Anova) followed by Bonferroni's multiple comparison test. A *P *value less than 0.05 (*) or 0.01 (**) was considered to be statistically significant. For quantification of cell type-specific marker expression, at least 1000 cells in three independent cultures, respectively, were counted in duplicates for each time point and were analyzed by unpaired t-test.

Data of spikes/minute and kappa values were analyzed by paired t-test. Data are presented as mean ± standard error of means, *n *refers to the number of recordings. All data analyses have been performed with GraphPadPrism, version 4.0.

## Results

### nSFEB-derived migrating progeny consists of a mixed neural cell population

**Figure 1:**
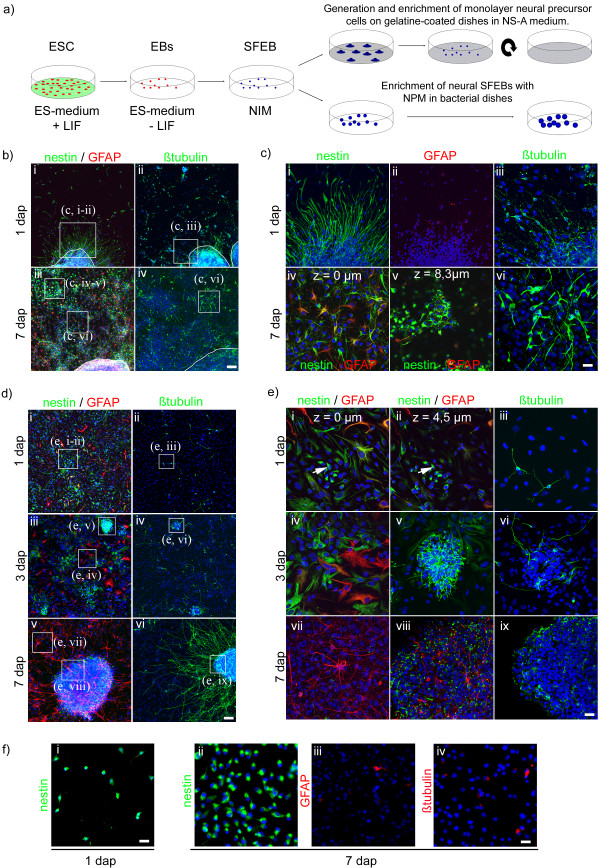
**Phenotype of nSFEB-, SNP- and MNP-derived neural cell populations**. Schematic illustration of the generation of nSFEBs and MNPs from ES cells (**a**). Low magnification overview **(b; d) **and regions of interests (ROIs) presented at higher magnifications **(c; e; f) **with nestin^+^, GFAP^+ ^or βtubulin^+ ^cells in adherent nSFEB **(b; c)**, SNP **(d; e) **or MNP **(f) **cultures at different time points indicated by dap (days after plating) are given. Adherently growing nSFEB aggregates are delineated by a white line (**b**). White boxes indicate ROIs in **b **and **d **which were presented at higher magnification in **e **and **c **(brackets indicate the corresponding ROI). In **c, iv + v **and **e, i + ii **ROIs are illustrating the same region at different z-levels (the z-level given in μm), respectively.

In contrast to mere neural selection protocols [16], we primarily pre-differentiated ES cells by neural induction. For this, embryoid bodies were maintained in ES cell medium without LIF for only one day before cells were transferred to the neural induction medium (NIM) (Figure 1a). After 7 days in NIM, serum-free, floating cultures of embryoid body-like aggregates (SFEBs) had developed which predominantly consisted of neural stem and progenitor cells as well as residual undifferentiated ES cells [13,19-21]. For further purification, SFEBs were cultivated as free-floating aggregates in neural proliferation medium (NPM) under the influence of EGF and FGF-2 and passaged at least 2-3 times whereupon neural precursor cell-enriched SFEBs (nSFEBs) had developed.

In accordance to previous publications [13,21], one day after plating nSFEBs on laminin coated substrates, two different cell populations were observed that migrated radially, leaving the edges of the adherent nSFEB aggregates. The first population displayed an elongated-flat morphology and most of these cells were nestin^+^/GFAP^-^, while only very few of them were nestin^-^/GFAP^+ ^(Figure 1b, c). The second population was bipolar and round shaped and predominately βtubulin^+ ^(Figure 1b, c).

Seven days after plating, the elongated-flat cell population had migrated a considerable distance away from the edges of the nSFEB aggregates and generated a flat cell layer of nestin^+^/GFAP^-^, nestin^+^/GFAP^+ ^and nestin^-^/GFAP^+ ^cells (Figure 1b, c). Interestingly, in addition to the flat cell layer and the βtubulin^+ ^population (Figure 1c, iv and 1vi), secondary cell clusters appeared on top of the flat cell layer (Figure 1c, v). These secondary clusters consisted of round nestin^+^/GFAP^- ^cells that were smaller than the elongated-flat cells. In addition, the nestin immunoreactivity in round nestin^+^/GFAP^- ^cells was considerably stronger in comparison to nestin^+^/GFAP^- ^and nestin^+^/GFAP^+ ^elongated-flat cells (Figure 1b, c).

After further cultivation in the presence of growth factors, secondary clusters of round nestin^+^/GFAP^- ^cells gave rise to larger structures that we termed secondary neural aggregates (Figure 1d, v, vi). They comprised βtubulin^+ ^neurons, nestin^+^/GFAP^- ^cells and nestin^-^/GFAP^+ ^astrocytes (Figure 1e, viii, ix). The flat cell layer of migrating elongated cells and the secondary neural clusters and aggregates were referred to as the secondary neural cell population (SNP), which consisted of a mixed neural phenotype. Most importantly, we and others [21] were not able to detect contractile tissue indicative for mesodermal differentiation or epithelia like cells indicative for endodermal differentiation within the SNP during the first 7 days after plating nSFEBs under the influence of FGF-2 or EGF/FGF2 (data not shown). Residual non-neural cells, however, were detected within nSFEBs and also within the SNP at later time points (>14 days after plating) during terminal differentiation (data not shown).

### The secondary neural cell populations (SNPs) derived from plated nSFEBs can selectively be harvested

To isolate SNPs derived from primary nSFEB aggregates on a laminin substrate at early time points and thus to eliminate non-neural cells determined to become, for instance, contractile tissue or epithelial-like cells, we first detached the entire cell population including primary nSFEB aggregates and SNPs and separated those populations by gentle trituration and sedimentation of nSFEB aggregates. The SNPs were then again dissociated and plated on a laminin coated substrate and characterized by immunocytochemistry and MEA recordings.

One day after plating dissociated SNPs, a mixed neural cell population could be observed which consisted of elongated-flat nestin^+^/GFAP^-^, nestin^+^/GFAP^+^, nestin^-^/GFAP^+ ^cells and processes-bearing βtubulin^+ ^cells (Figure 1d, e). Already at this early time, we again observed secondary clusters similar to those found in nSFEB cultures. They consisted of small round nestin^+^/GFAP^- ^cells which were located on top of the elongated-flat neural cells (Figure 1e, i, ii). Three days after plating dissociated SNPs, secondary clusters adopted a rosette-like structure composed of nestin^+^/GFAP^- ^and βtubulin^+ ^cells with radially extending neurites. Interestingly, secondary clusters did not exhibit GFAP^+ ^cells yet (Figure 1e, v + vi). Seven days after plating dissociated SNPs, secondary clusters gave rise to larger secondary neural aggregates, similar to those observed within nSFEB cultures. They were composed of nestin^+^/GFAP^-^, nestin^-^/GFAP^+ ^cells and, particularly at the edges, βtubulin^+ ^neurons (Figure 1e, viii + ix). At the same time, the cell population in between secondary clusters or aggregates comprised nestin^-^/GFAP^+ ^flat cells (Figure 1d, v; 1e, vii) and βtubulin^+ ^neurons (Figure 1d, ii, iv; e, iii). Importantly, all independently predifferentiated SNP cultures, no formation of contractile tissue was observed.

### MNPs consist of a pure radial glia-like cell population

In contrast to neural aggregate cultures (nSFEB or SNP), MNPs were cultivated on gelatin-coated substrates in neural stem cell medium (NS-A) supplemented with EGF and FGF-2 with 2-3 passages per week (Figure 1a). As revealed by immunocytochemical analyses, all MNPs were homogeneously nestin^+ ^(Figure 1f) Pax6^+^/Vimentin^+^/RC2^+ ^(data not shown). After cultivation of MNPs for 7 days on a laminin substrate under the influence of FGF-2, few cells expressed GFAP or βtubulin (Figure 1f, ii-iv). This indicated that the gelatin substrate in combination with EGF and FGF-2 was able to keep MNPs in an immature radial glial state and that a laminin substrate together with FGF-2 alone was not able to completely prevent differentiation.

### nSFEB- and SNP-, but not MNP-derived cells are able to generate synchronously oscillating neuronal networks

**Figure 2:**
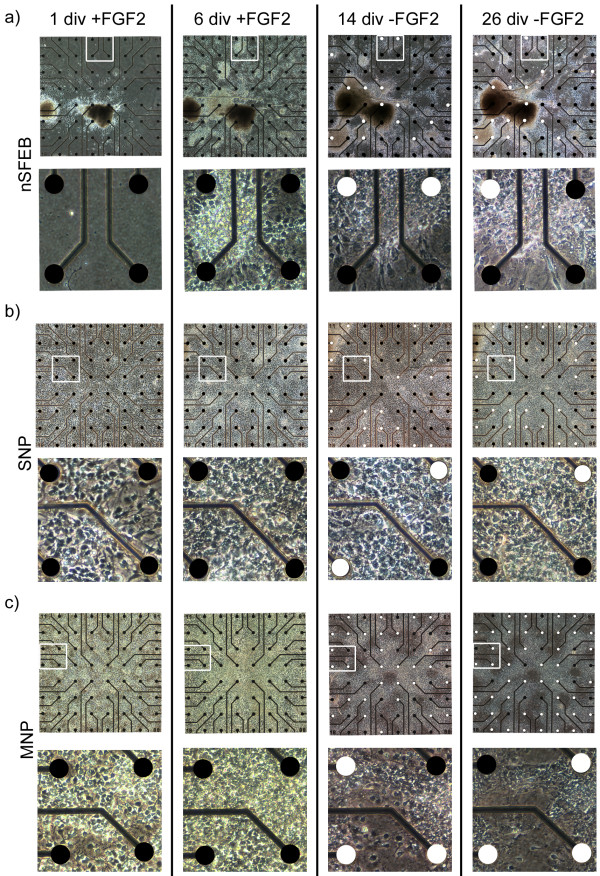
**Development of neuronal activity of nSFEB, SNP or MNP cultures**. Overview **(a; b; c; upper panel) **and detailed **(a; b; c; lower panel) **phase-contrast images of different cultures on MEAs in the presence (+FGF2) and after the removal (-FGF2) of FGF2. White-labeled electrodes represent electrodes that detect spontaneous spikes and black-labeled electrodes detect no spikes. White boxes in upper panels indicate regions which are presented at higher magnification in the lower panels. Electrode diameter = 30 μm.

**Figure 3:**
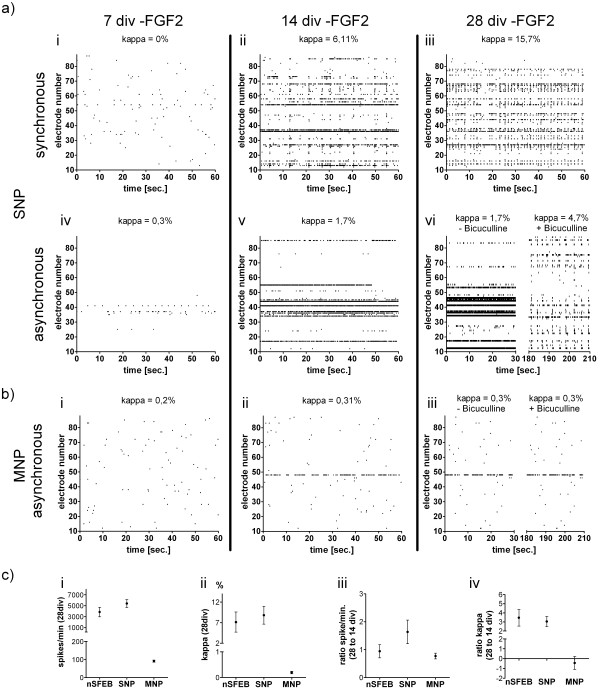
**Spike raster plots (SRPs), mean activity levels and kappa values**. Examples of SRPs and corresponding kappa values illustrate the temporal and spatial development of spikes detected by electrodes (vertical axis) over a time period of 60 seconds (horizontal axis) in different cultures (**a**, **b**) and different time points after the removal of FGF2. The effect of 50 μM bicuculline is illustrated in right panels of **a, vi **and **b, iii**. Mean activity levels (spikes/minute) and the degree of synchronous bursting (kappa values) at 28 div are illustrated for different cultures (**c**, **i + ii**). The development of mean activity levels and synchronous bursting between 14 and 28 div is given as ratios (**c**, **iii + iv**).

To determine if nSFEB-, SNP- and MNP-derived neural cells are able to generate functional neuronal networks, we plated nSFEBs (5-10 aggregates), SNPs or MNPs (4-8 × 10^4 ^cells/100 μl) on microelectrode arrays (MEAs). After a proliferation period of about one week under the influence of FGF-2, this growth factor was gradually removed to initiate terminal differentiation (Figure 2). After withdrawal of FGF-2, MEA recordings were performed every 2 to 3 days over a time period of 4-12 weeks. Differentiation without FGF-2 for at least 14 days led to the appearance of active (spike detecting) electrodes in all different approaches (Figure 2a-c). In accordance with previously published results [11], the functional development of nSFEB- or SNP-derived neuronal networks could be divided into three distinct stages (Figure 3). Since MEA recordings of nSFEB and SNP cultures revealed no detectable differences between these two cultures, we only exemplarily illustrated spike raster plots (SRP) of SNP cultures. During the first week after initiating the differentiation, spontaneous spiking activity was observed (stage 1; Figure 3a, i + iv; b, i). During the second week after initiating the differentiation, also spikes organized in trains or bursts were detected on an increased number of MEA electrodes (stage 2). From 2-4 weeks onwards after initiating the differentiation, the activity patterns of nSFEB and SNP cultures either progressed towards synchronously oscillating bursts (stage 3, Figure 3a, ii + iii) or remained unsynchronized within stage 2 (Figure 3a, v + vi). Some nSFEB or SNP cultures that did not spontaneously progress towards synchronous stage 3 activity were then treated with bicuculline, a GABA-A receptor antagonist which is known to elicit synchronous stage 3 network activity in previously non-synchronously active stage 2 populations provided morphological prerequisites are given. Interestingly, bicuculline robustly converted non-synchronous stage 2 activity towards synchronous stage 3 activity in all 11 independent nSFEB and SNP-derived neuronal cultures (Figure 3a, vi).

We then verified the functional activity of MNP-derived neuronal populations. While electrodes abundantly detected spontaneous spike activity and trains of spikes commencing 14 days after initiating the differentiation, synchronous bursts were absent during an observation time of up to 3 months in 20 independent MEA cultures (Figure 3c). Importantly, also the application of bicuculline did not elicit synchronous network activity in MNP-derived neuronal populations (Figure 3b, iii). As also the seeding density of neuronal populations is known to be of importance with respect to functional network generation [7,24,25] we plated nSFEB-, SNP- and MNP-derived neural cell populations at varying seeding densities. Similar to primary tissue-derived neuronal networks [7,24,25] higher seeding densities of nSFEB or SNP cultures changed the development of synchronous neuronal networks. However, varying seeding densities did not improve the functional activity of MNP-derived cultures.

Comparison of mean activity levels (spikes per minute) and degrees of network synchrony (kappa values) of nSFEB-, SNP- and MNP-derived neuronal networks revealed major differences. Twenty-eight days after initiation of differentiation, the mean spike rates in nSFEB or SNP cultures were 42-fold or 52-fold higher, respectively, than in MNP cultures (Figure 3c, i). Furthermore, kappa values at this point in time were clearly indicative of synchronous network activity in nSFEB and SNP cultures. Kappa values in MNP cultures were expectedly low suggesting asynchronous activity. This is in line with the comparably low overall activity levels of MNP cultures that were hardly consistent with synchronous bursting (Figure 3c, ii).

To measure developmental changes of network activity between 14 and 28 days after initiating the differentiation, spike rate ratios (spike rates at 28 days/14 days) or kappa ratios (kappa values at 28 days/14 days) were calculated for nSFEB-, SNP- or MNP-derived network activity. While spike rate ratios revealed no major differences between groups, kappa ratios suggested an increase in synchrony of network activity merely of nSFEB or SNP cultures (Figure 3c, iii + iv).

### Percentages of active electrodes in nSFEB- and SNP-derived populations are regionally different

An overview of the distribution of active electrodes in different ES cell-derived neural population is given in figure 2 with active electrodes depicted in white and inactive electrodes in black. In the present study, most electrodes that detected spike activity (active electrodes) recorded multiphasic spikes, suggesting a somatic origin of those signals [4].

As nSFEB and SNP cultures exhibited a heterogeneous spatial distribution of cells with primary nSFEB aggregates (only in nSFEB cultures), secondary neural aggregates (in nSFEB and SNP cultures) and the interjacent cell population consisting of a flat cell layer (Figure 2), we accordingly analyzed the spatial distribution of active electrodes which were participating in synchronous burst activity.

We observed that most active electrodes were localized at the edges of primary nSFEB or secondary neural aggregates (edges: 75% ± 14.4% vs. centers: 7.2% ± 7.4%, p < 0.001) and that fewer active electrodes were localized within the interjacent cell population (interjacent cell population: 25.8% ± 10.6%).

### Neural aggregate and MNP cultures contain considerable numbers of neurons and glial cells

**Figure 4:**
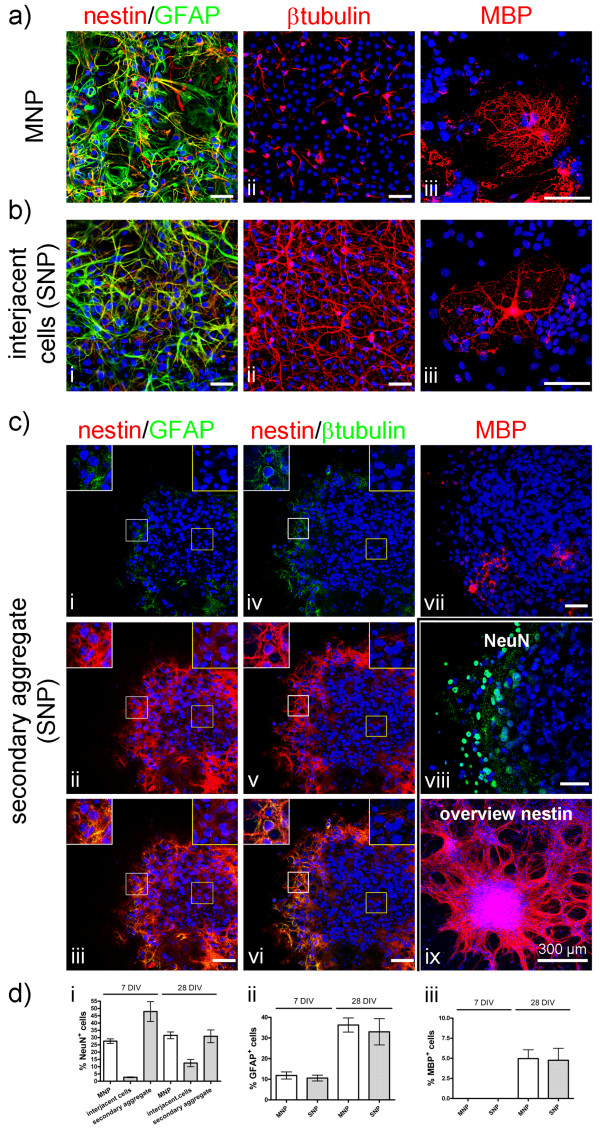
**Neuronal and glial differentiation of neural aggregate and MNP cultures**. Images illustrate the composition of MNP or neural aggregate (nSFEB or SNP) cultures **(a-c)**. Both cultures comprise nestin^-^/GFAP^+^, nestin^+^/GFAP^- ^and nestin^+^/GFAP^+ ^**(a, i; b, i; c i-iii)**, βtubulin^+ ^**(a, ii; b, ii; c, iv-vi) **and MBP^+ ^cells **(b, iii; c, iii; d, vii)**. Optical slices (1 μm) illustrate the morphology and localisation of nestin^+^, βtubulin^+^, GFAP^+^, NeuN^+ ^and MBP^+ ^cells within the core (yellow boxes) or edge (white boxes) region of secondary neural aggregates (**c, i-viii**). Regions of interest indicated by boxes are illustrated at higher magnifications **(c, i-vi)**. Note, for visualisation of MBP^+ ^multi-branched oligodendrocytes a set of optical slices were stacked (**c, vii**). Low-magnification image of nestin^+ ^cells in neural aggregate cultures containing secondary neural aggregates and the interjacent cell population (**c, ix**) Scale bar: 75 μm. Diagrams illustrate percentages of NeuN^+ ^neurons **(d, i)**, GFAP^+ ^astrocytes **(d, ii) **or MBP^+ ^oligodendrocytes **(d, iii) **within neural aggregate or MNP cultures at the indicated days *in vitro *(div).

To analyze if the differences in mean activity levels and kappa values detected between neural aggregate cultures (nSFEB or SNP) and MNP cultures were caused by varying numbers of neurons, astrocytes or oligodendrocytes, we quantified total numbers of these cell types within neural aggregate or MNP cultures at 7 or 28 days after initiating the differentiation (Figure 4). Since neural aggregate cultures comprised neural aggregates and interjacent cells, we analyzed these cultures accordingly. To quantify different cell types within a fairly pure population of neural cells, we spared analyzing primary nSFEB aggregates as several of these heterogeneous structures contained considerable numbers of non-neural cells like e.g. cardiomyocytes or epithelial cells which rendered a stringent quantification impossible. Since cell type-specific markers like βtubulin, GFAP or MBP detect cytoplasmic structures and cellular processes, it was not possible to reliably quantify cells positive for those markers within densely packed secondary neural aggregates even by laser scanning microscopy. Quantification of those markers in MNP cultures or within the interjacent cell population in neural aggregate cultures was reliable. Nevertheless, to quantify at least neuronal numbers within secondary aggregates, we applied NeuN immunocytochemistry which specifically detects neuronal nuclei. By means of this marker, also densely packed cells within secondary neural aggregates could reliably be quantified. For better comparison of the quantitative results, NeuN was applied as the quantitative neuronal marker in different groups instead of βtubulin.

Both MNP and neural aggregate cultures comprised nestin^-^/GFAP^+^, nestin^+^/GFAP^-^, and nestin^+^/GFAP^+ ^cells with diverse morphology, process-bearing βtubulin^+ ^neurons and multi-branched MBP^+ ^oligodendrocytes at 28 days after the initiation of differentiation (Figure 4a, b). After initiating the differentiation by gradual withdrawal of FGF-2, the homogeneous population of radial glia-like MNPs differentiated into equally distributed βtubulin^+ ^neurons, GFAP^+ ^astrocytes and MBP^+ ^oligodendrocytes (Figure 4a). Laser scanning microscopy revealed that secondary neural aggregates comprised a core and edge region (Figure 4c). The core region was composed of mainly nestin^+ ^cells (Figure 4c, i-vi; yellow box), whereas edges appeared to harbour higher numbers of GFAP^+^, βtubulin^+ ^and few MBP^+ ^cells (Figure 4c, i-vi; white box).

Seven days after initiating the differentiation, 27.52% ± 1.547% of all MNP-derived cells were NeuN^+ ^neurons. The number of neurons remained stable until 28 days after initiating the differentiation (31.52% ± 2.340%, Figure 4d, i).

Numbers of NeuN^+ ^neurons in neural aggregate cultures were separately quantified within secondary neural aggregates and interjacent cells. Similar to the distribution of βtubulin^+ ^cells, we could barely find any NeuN^+ ^cells within centers of secondary aggregates, in comparison to the edges where we found 47.9% ± 6.8% NeuN^+ ^neurons at 7 days after initiating the differentiation (Figure 4c, viii). At 28 days after initiating the differentiation, percentages of NeuN^+ ^cells tended to decrease at the edges towards 30.8% ± 4.4% (Figure 4d, i). The interjacent cells in between secondary aggregates contained only 2.7% ± 0.3% NeuN^+ ^neurons at 7 days after initiating the differentiation. After 28 days, percentages of NeuN^+ ^neurons tended to increase towards 12.5% ± 2.5% (Figure 4d, i). This quantitative constellation suggests either a dynamic migration of cells between aggregates and the interjacent area or ongoing neurogenesis.

Considerable numbers of nestin^+ ^cells within the centers of secondary aggregates indicate that antibodies do have access to this dense cellular structure (Figure 4c ii, v, iii, vi).

Thus, both neural aggregate (nSFEB or SNP) and MNP cultures contained considerable numbers of neurons. While NeuN^+ ^neurons in MNP cultures were evenly distributed, NeuN^+ ^neurons in neural aggregate cultures were predominantly located at the edges of secondary neural aggregates and to a lower percentage within the interjacent population. However, also those regions within neural aggregate cultures that showed lower neuronal percentages (interjacent population) were found to be functionally integrated within synchronous network activity (see above), suggesting that not only the mere number of neurons within cultures is responsible for different degrees of electrophysiological activity.

As it is known that astrocytes are able to regulate synaptogenesis, neurite outgrowth and neuronal maturation [26,27], and that myelinating oligodendrocytes impact on action potential propagation, we also analyzed the numbers of GFAP^+ ^astrocytes and MBP^+ ^oligodendrocytes within neural aggregate and MNP cultures. As described above, GFAP^+ ^or MBP^+ ^cells were found predominantly within the interjacent cell population and at the edges of secondary neural aggregates (Figure 4b, iii; c, i, vii). While a reliable quantification of GFAP^+ ^or MBP^+ ^cells within the densely packed edges of secondary aggregates was not possible, we found that the interjacent cells showed no significant differences in percentages of GFAP^+ ^astrocytes or MBP^+ ^oligodendrocytes in comparison to MNP cultures at 7 or 28 days after initiating the differentiation (Figure 4d, ii). In contrast to MNP cultures, the distribution of cells within neural aggregate cultures was not homogeneous limiting the validity of a quantitative comparison.

### Neural aggregate and MNP cultures differentiate into neurons positive for GABA or vGlut2

As differentiation into inhibitory GABAergic and excitatory glutamatergic neurons represents a prerequisite for the generation of functional neuronal networks, we immunocytochemically verified the expression of GABA and vGlut2 in neural aggregate (nSFEB or SNP) and MNP cultures after 28 days of differentiation.

**Figure 5:**
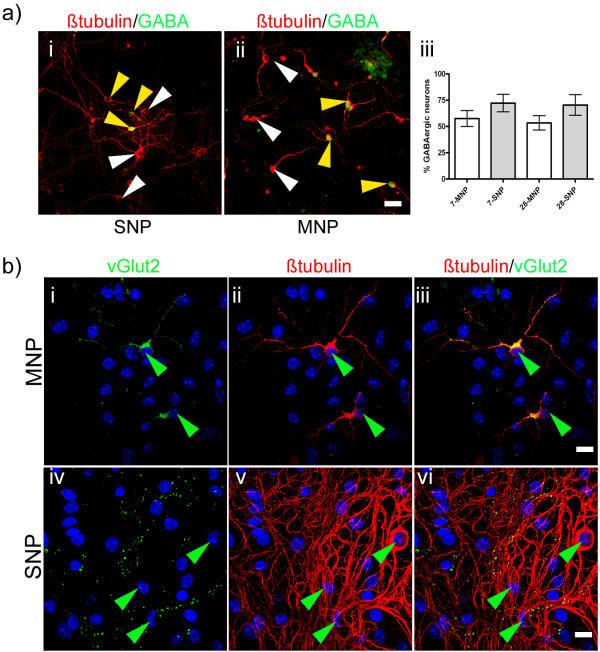
**Morphology of GABA^+^- and vGlut2^+^-neurons in neural aggregate or MNP-cultures**. Examples of the morphology of non-GABAergic **(white arrow heads) **and GABAergic **(yellow arrow heads) **neurons **(a) **and the sub-cellular localization of vGlut2 **(b) **in MNP- **(a, ii; b, i-iii) **and neural aggregate-derived **(a, i; b, iv-vi) **βtubulin^+^neurons **(green arrow heads) **at 28 days after initiating the differentiation. Quantification of GABA^+^/βtubulin^+ ^cells 7 and 28 days after initiating the differentiation **(a, iii) **Note, the intensive βtubulin^+ ^cell labeling in neural aggregate cultures stems from the dense neurite network. Scale bar = 20 μm (**a**) and 10 μm (**b**).

GABA^+ ^neurons were found in both neural aggregate and MNP cultures. However, while GABA^+ ^neurons in neural aggregate cultures extended long and ramified neurites, GABA^+ ^neurons within MNP cultures exhibited shorter and less ramified neurites (Figure 5a, i + ii). Quantification of GABAergic neurons within neural aggregate or MNP cultures, at 7 or 28 days after initiating the differentiation, showed no significant differences (Figure 5a, iii). In neural aggregate cultures, GABAergic neurons were predominantly found at the edges of neural aggregates and within the interjacent cell population.

In neural aggregate cultures, we found the typical punctuated vGlut2 staining localized on βtubulin^+ ^neurites, a distribution which is known to correspond to vesicular glutamate transporters on synapses. However, vGlut2 labeling in differentiated MNP cultures was localized cytoplasmically (Figure 5b). Due to the different cellular localization of vGlut2^+ ^structures in both groups, a reliable quantification of mature glutamatergic neurons that obviously do not exist within MNP cultures was not possible.

### Neural aggregate and MNP cultures reveal differences in neurite morphology

**Figure 6:**
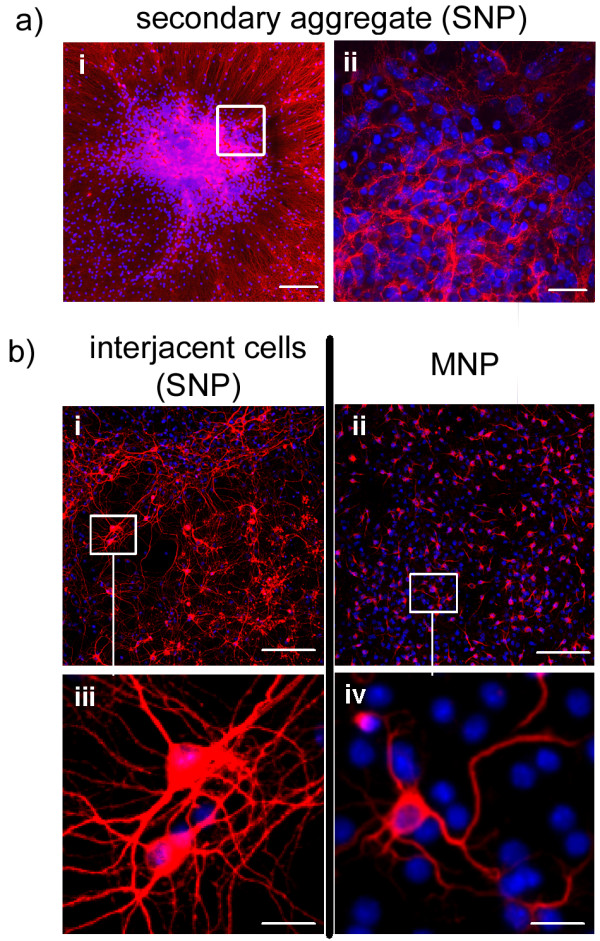
**Morphology of neurons within neural aggregate and MNP cultures**. Distribution of βtubulin^+ ^neurons within secondary neural aggregates **(a; b, i + iii) **and MNP cultures **(b, ii + iv)**. Two weeks after the initiation of differentiation, neural aggregate-derived βtubulin^+ ^neurons are located within secondary aggregates (**a **overview: **i**, stacked set of optical slice at the edge indicated by white box in i: **ii) **and on top of flat-elongated interjacent cells **(b, i + iii)**. Higher magnifications of neural aggregate- **(b, iii) **and MNP- **(b, iv) **derived βtubulin^+ ^neurons reveal differences in neurite length and morphology. Scale bar = 100 μm **(a, i; b, i + ii)**; 20 μm **(a, i; b, iii + iv)**.

Furthermore, differences in neurite morphology between neural aggregate on the one hand and MNP cultures on the other were detected (Figure 6).

While βtubulin^+ ^neurons within neural aggregates (Figure 6a, i) and within the interjacent cell population (Figure 6a, ii; b, i + iii) of neural aggregate cultures (nSFEB or SNP) developed a dense network of neurites as soon as 14 days after initiating the differentiation, MNP-derived neuronal populations were not able to generate a morphologically dense network of neurites (Figure 6b, ii + iv).

## Discussion

We detected functional discrepancies between different ES cell-derived neuronal populations by using the MEA technology: (i) a population of homogeneous, radial glia-like cells [13] termed "monolayer neural precursors" (MNP), and (ii) a heterogeneous neural cell population comprising neural stem/progenitor cells, committed neural cells as well as mature cells like neurons and glial cells termed "secondary neural cell population" (SNP). Previous studies have demonstrated that ES cell-derived heterogeneous neural cell populations cultivated as neurospheres or as adherently growing cultures are unstable and alter their differentiation potential with time by adopting a gliogenic fate after few passages [16,28]. Whether this phenomenon is mediated by an intrinsic program or rather by the environment consisting of more committed neural cells which serve as a niche to induce enhanced glial maturation is still under debate. It was an important step forward when new protocols were introduced allowing to generate stable ES cell-derived homogeneous radial glia-like cells which can be enriched in nearly unlimited numbers as niche-independent, adherently growing neural stem cells without losing their neurogenic fate [13]. However, we demonstrate that neurons derived from homogeneous, radial glia-like MNPs were only able to show spontaneous spike activity without generating synchronously oscillating neuronal networks. In contrast, neurons derived from heterogeneous SNPs reliably generated functional network activity with synchrony even increasing with age. Interestingly, both neural cell populations were initially generated from serum-free, floating cultures of embryoid body-like aggregates (SFEBs) before cells were split in accordance with the two different protocols. This suggests that the subsequently applied cell culture procedures were responsible for the functional differences.

### MNP- but not SNP-derived neurons remain within immature neuronal stages

As both neural aggregate- and MNP-cultures differentiated into sufficient numbers of neurons, astrocytes and oligodendrocytes, the mere quantity of those cells appeared not to be responsible for differences in functional network generation. We could demonstrate that electrophysiological network activity was not limited to the edges of neural aggregates that comprised ~31% mature NeuN^+ ^neurons at 28 days after initiating the differentiation. Indeed, electrodes situated outside neural aggregates also participated in network activity although the interjacent cell population situated here contained only ~13% mature NeuN^+ ^neurons. Thus, neuronal percentages amounting to approximately 32% within dense MNP cultures at 28 days after initiating the differentiation are indicative of other causes for the missing network activity such as impaired neuronal maturity. This assumption is substantiated by the following observations.

Our morphological analyses suggest that MNP-derived neurons in general seem to remain within an immature stage as they sent out only sparsely ramified and short neurites despite a differentiation period for up to 3 months. Such delayed maturation is in accordance with patch-clamp experiments demonstrating that in MNP-derived neurons single action potentials cannot be evoked until 30 days of differentiation [13] while aggregate-based ES cell-derived neurons exhibit evoked repetitive firing already after 12 days of differentiation [17,29,30]. Furthermore, in contrast to SNP-derived neurons which exhibited a punctuated pre-synaptic vGlut2 labeling on βtubulin^+ ^neuronal processes, in line with a mature neuronal morphology, we demonstrated that MNP-derived neurons showed a cytoplasmic vGlut2 expression similar to immature embryonic neurons *in vivo *[31]. A proper quantification of glutamatergic neurons in different groups by means of this marker was not possible as vGlut2 antibodies labeled presynaptic structures in aggregate cultures and cytoplasmic structures in MNP cultures preventing an acceptable comparison. It has to be determined in future studies by direct labeling of glutamate, if a reliable quantification of glutamatergic neurons within aggregate or MNP cultures is feasible. However, this approach has to take into consideration that, particularly in neural precursor populations, glutamate might be of metabolic, neurotransmitter or even precursor origin [32,33]. Our morphological analyses specifically of GABAergic neurons also suggested an immature phenotype in MNP-derived cultures while aggregate-derived cultures exhibited GABAergic neurons of mature morphology with ramified and long neurites. The mere quantities of GABAergic neurons, however, were similar in both cultures. Thus, as network activity depends on the presence of mature excitatory and inhibitory neurons, our morphological results are in line with impaired neuronal network function. To further examine neuronal populations with respect to the functional balance of inhibitory or excitatory neurotransmitter systems, we pharmacologically modulated network activity and observed that the inhibition of GABAergic transmission in neural aggregate cultures (nSFEB or SNP) transformed asynchronous stage 2 activity into synchronous stage 3 activity, whereas MNP-derived neuronal populations remained within asynchronous stages. This functional constellation also suggests that MNP cultures miss morphological prerequisites supporting synchronous network activity.

### SNPs represent a heterogeneous neural cell population with the ability to generate functional neuronal networks in vitro

In accordance with previous studies, we demonstrated that pure neural cell populations migrating away from neural aggregate edges on a laminin substrate can be found only within the first 7 days after attachment of nSFEBs. This period of fairly pure neural cell migration is followed by non-neural cells or residual ES cells that also migrate away from nSFEBs after further adherent cultivation [13,21]. This observation is in line with reports of residual Oct-4^+ ^cells and non-neural cells within nSFEB aggregates [19-21]. To purify neural populations, we selectively harvested migrating cells within the first week after attachment of nSFEBs by applying a sedimentation technique and termed this purified population the secondary neural cell population (SNP). We demonstrated that dissociated SNPs gave rise to neural populations that (i) were devoid of residual ES cells indicated by negative Oct-4 immunocytochemistry (data not shown), contractile tissue and epithelial-like cells in more than 130 cultures from three independently pre-differentiated ES cell populations, and (ii) generated functional neuronal network activity.

Interestingly, we showed that round nestin^+^/GFAP^- ^cells, which were found in the periphery of adherently growing nSFEBs a few days after attachment, reorganized to neural rosette-like structures on top of a cellular monolayer consisting of nestin^+^/GFAP^-^, nestin^+^/GFAP^+ ^and nestin^-^/GFAP^+ ^cells. Thereby, the cellular monolayer might contain mixed types of neural precursors and astrocytes which might serve as a neurogenic niche for neural rosette-forming nestin^+^/GFAP^- ^cells similar to postnatal astrocytes [34,35]. This suggests that SNP-derived neural rosettes comprised neuroepithelial cells displaying several *in vivo *properties and giving rise to neural aggregates [36-40]. Laser-scanning microscopy revealed that only the edges of neural aggregates comprised higher numbers of mature neural cells (NeuN^+ ^neurons, GFAP^+ ^astrocytes or MBP^+ ^oligodendorcytes) while aggregate centres predominantly contained immature nestin^+ ^neural cells. In agreement with this, neural aggregates revealed low electrophysiological network activity within their centres (~7% of all electrodes) and high network activity at their edges (~75% of all electrodes). The development from neuroepithelial cells organised in rosette-like structures towards neural aggregates might lead to a hypothesis that immature neural cells remain within the centre of neural aggregates, virtually constituting the niche of functional neuronal progeny.

In previous studies, we demonstrated that a prolonged cultivation of ES cell-derived neural cell populations under the influence of FGF-2 resulted in the formation of substrate-adherent ES cell-derived neural aggregates (SENAs) that appeared to be similar to those secondary neural aggregates that were described in the present study. Interestingly, SENAs did not form teratomas after transplantation into an animal model of Huntington's disease [15]. We hypothesize that both, SENAs and secondary neural aggregates, can be derived from those neural rosettes by different protocols [15]. If secondary neural aggregates are also devoid of teratoma forming cells remains to be verified.

### Do MNPs exhibit intrinsically impaired neurogenesis or miss the right environment for proper neuronal maturation?

Interestingly, we also found clustering of MNP cells at higher seeding densities, but those were devoid of rosette formation, showed an even distribution of mature and immature cells and contained only short-ramified neurons after the initiation of differentiation. It remains to be determined, if these radial glia-like cells are able to organize as neural rosettes in co-culture with astrocytes [34,35] or if they have lost the ability to recapitulate early neural stage development resulting in a restricted developmental potential. A possible reason for inhibited maturation in gelatine-cultivated monolayer precursors might also be shortcomings in the expression of extracellular matrix (ECM) molecules in comparison to the heterogeneous neural cell populations. Recent reports demonstrated that different ECM molecules such as fibronectin and laminin exert strong influences on ES cell-derived neural stem and progenitor cells resulting in the expression of different transcription factor profiles, as well as diverse morphologic and electrophysiological properties of their differentiated mature neuronal progeny [29]. Furthermore, as different subclasses of GFAP^+ ^cells are known to considerably support the electrophysiological maturation of neurons [27] and enhance or inhibit neurite outgrowth [26], it might be speculated that the different types of GFAP^+ ^cells which we found at early stages of neural aggregate development but not within homogeneous MNP cultures are important for network function. Thus, niche-independent neural development might provide a high degree of cellular homogeneity at the cost of the proper milieu to generate functionally mature neurons *in vitro*. This speculation of course will have to be substantiated by additional investigations. As the ability of precursor-derived neurons to form functional neuronal networks represents a prerequisite to properly restore functional deficits in succession to neuronal degeneration, our data are directly linked to the field of regenerative medicine.

### The MEA technology can be used to unmask functional differences of neural precursors

The data presented here demonstrates that the MEA technology detects functional differences in neural precursor cell development that exceed the functional verification of single neurons. So far, functional neuronal analyses by intracellular recordings have shown that conventional ES cell-derived neural precursors differentiate into electrophysiologically active neurons [13,29,41]. We confirmed this by applying the MEA technology to ES cell-derived neuronal networks, suggesting that the description of synchronous network activity represents a functional feature that should be additionally taken into consideration in order to characterize properties of precursor-derived neuronal populations. Furthermore, it could be shown that multi-site extracellular recordings were able to spatially resolve functional differences within individual neural cultures that developed compact, three-dimensionally arranged cellular aggregates as well as the interjacent cellular monolayers that were evenly distributed in between those neural aggregates.

## Conclusion

Our investigations provide evidence that ES cell-derived neural precursors derived from heterogeneous neural aggregates give rise to neurons that are able to generate functional neuronal networks between 2-4 weeks after initiating the differentiation *in vitro*, whereas neurons derived from a homogeneous monolayer of radial glia-like cells do not exceed the stage of uncorrelated spontaneous activity during a differentiation period of up to 12 weeks under the cell culture conditions used in the present study. If MNP-derived neuronal populations adopt a functional state under modified cell culture protocols *in vitro *or after transplantation *in vivo *remains to be determined. Nonetheless, our findings indicate that niche- and aggregate-based differentiation clearly improves the functionality of ES cell-derived neural populations *in vitro*.

## Authors' contributions

SI participated in conceiving, conception and designing the study, carried out the acquisition, analysis, interpretation of data and drafted the manuscript. ST participated in the interpretation of data, performed the statistical analysis and critically revised the manuscript for important intellectual content. HPH and MS critically revised the manuscript for important intellectual content. MD coordinated and conceived the study, participated in its design and in the interpretation of data and critically revised the manuscript for important intellectual content. All authors read and approved the final version of the manuscript.
